# Interventions to improve the rate or timing of initiation of antiretroviral therapy for HIV in sub-Saharan Africa: meta-analyses of effectiveness

**DOI:** 10.7448/IAS.19.1.20888

**Published:** 2016-08-08

**Authors:** Matthew P Fox, Sydney Rosen, Pascal Geldsetzer, Till Bärnighausen, Eyerusalem Negussie, Rachel Beanland

**Affiliations:** 1 Department of Global Health, Boston University, Boston, MA, USA; 2 Health Economics and Epidemiology Research Office, Department of Internal Medicine, School of Clinical Medicine, Faculty of Health Sciences, University of the Witwatersrand, Johannesburg, South Africa; 3 Department of Epidemiology, Boston University School of Public Health, Boston, MA, USA; 4 Department of Global Health and Population, Harvard T.H. Chan School of Public Health, Boston, MA, USA; 5 Africa Health Research Institute, Somkhele, South Africa; 6 Institute for Public Health, University of Heidelberg, Heidelberg, Germany; 7 Department of HIV/AIDS, World Health Organization, Geneva, Switzerland

**Keywords:** retention, attrition, interventions, systematic review, meta-analysis, linkage, art initiation

## Abstract

**Introduction:**

As global policy evolves toward initiating lifelong antiretroviral therapy (ART) regardless of CD4 count, initiating individuals newly diagnosed with HIV on ART as efficiently as possible will become increasingly important. To inform progress, we conducted a systematic review of pre-ART interventions aiming to increase ART initiation in sub-Saharan Africa.

**Methods:**

We searched PubMed, Embase and the ISI Web of Knowledge from 1 January 2008 to 1 March 2015, extended in PubMed to 25 May 2016, for English language publications pertaining to any country in sub-Saharan Africa and reporting on general adult populations. We included studies describing interventions aimed at increasing linkage to HIV care, retention in pre-ART or uptake of ART, which reported ART initiation as an outcome. We synthesized the evidence on causal intervention effects in meta-analysis of studies belonging to distinct intervention categories.

**Results and discussion:**

We identified 22 studies, which evaluated 25 interventions and included data on 45,393 individual patients. Twelve of twenty-two studies were observational. Rapid/point-of-care (POC) CD4 count technology (seven interventions) (relative risk, RR: 1.26; 95% confidence interval, CI: 1.02–1.55), interventions within home-based testing (two interventions) (RR: 2.00; 95% CI: 1.36–2.92), improved clinic operations (three interventions) (RR: 1.36; 95% CI: 1.25–1.48) and a package of patient-directed services (three interventions) (RR: 1.54; 95% CI: 1.20–1.97) were all associated with increased ART initiation as was HIV/TB service integration (three interventions) (RR: 2.05; 95% CI: 0.59–7.09) but with high imprecision. Provider-initiated testing (three interventions) was associated with reduced ART initiation (RR: 0.91; 95% CI: 0.86–0.97). Counselling and support interventions (two interventions) (RR 1.08; 95% CI: 0.94–1.26) had no impact on ART initiation. Overall, the evidence was graded as low or moderate quality using the GRADE criteria.

**Conclusions:**

The literature on interventions to increase uptake of ART is limited and of mixed quality. POC CD4 count and improving clinic operations show promise. More implementation research and evaluation is needed to identify how best to offer treatment initiation in a manner that is both efficient for service providers and effective for patients without jeopardizing treatment outcomes.

## Introduction

A persistent challenge confronting national HIV care and treatment programmes in low- and middle-income countries is late initiation of antiretroviral therapy (ART) and high patient attrition between HIV testing and treatment initiation. A recent systematic review found no significant change in CD4 cell counts at ART initiation in sub-Saharan Africa between 2002 and 2013, with the median remaining well below 200 cells/mm^3^-the original (and lowest) threshold for treatment eligibility [1]. The first published systematic review of retention in pre-ART care in sub-Saharan Africa estimated that 40% of patients testing positive for HIV were not linked to care to learn if they were eligible for treatment, and 30% who were eligible never started treatment [2]. Later systematic reviews have confirmed these findings of high rates of patient attrition before starting treatment despite eligibility under the prevailing threshold [3–5].

As global and national guidelines evolve toward initiating lifelong ART for all patients testing positive for HIV, regardless of CD4 cell count [6], the number of diagnosed patients who are not eligible for ART will diminish rapidly. The challenge of retaining patients in pre-ART care will lose its importance, to be replaced by the challenge of initiating on ART individuals newly diagnosed with HIV as efficiently as possible – in other words, maximizing the proportion of patients who do start treatment promptly, while minimizing the costs to both patients and the healthcare system. In recent years, a number of interventions have been developed and implemented that aim to increase uptake of ART for patients known or found to be eligible. To help inform continued progress in this area, we conducted a systematic review of the literature from 2008 to 2015 of pre-treatment interventions that reported the effect of the intervention on ART initiation in sub-Saharan Africa.

## Methods

This review is drawn from a larger systematic review of interventions to facilitate linkage to care and ART initiation conducted to support development of the World Health Organization's 2015 Consolidated Guidelines for the Use of Antiretroviral Drugs for Treating and Preventing HIV Infection and completed in June 2015. We include here the subset of articles in that review that were conducted in sub-Saharan Africa and reported rates and/or timing of ART initiation as an outcome.

### Search strategy and inclusion criteria

We included in the review randomized controlled trials, quasi-experimental trials, observational cohort studies and programme evaluations describing interventions to improve linkage to or retention in pre-ART care or to improve uptake of ART for those eligible. We searched for studies published or presented in English in 2008 or later pertaining to any country in sub-Saharan Africa and reported on general adult populations. Studies explicitly enrolling high-risk populations (e.g. sex workers) were excluded, as were those of interventions to improve initiation of ART for pregnant women in prevention of mother-to-child transmission programmes, as these comprise a different programmatic area than general HIV care. We limited the review to studies that included a comparison with standard of care (acknowledging that standard of care varies across settings), so that the effect size could be estimated and would be relevant to routine practice. We required that each study report an effect estimate for the intervention or risk/rates of outcomes between the two groups compared. Finally, as noted above, we required that each study report an outcome of an effect on the rate or timing of ART initiation. We accepted each article's own definition of “initiation” but presume that in nearly every case it referred to a patient being prescribed or dispensed an initial supply of ARVs.

To identify studies, we searched PubMed, Embase and the ISI Web of Knowledge from 1 January 2008 to 1 March 2015, for English language publications. Within each index, we combined “HIV” or “ART” with any of “linkage,” “pre-ART,” “initiation,” “retention,” “attrition,” “adherence,” “loss to follow-up” or “patient compliance” and any of “efficacy,” “evaluation,” “intervention” or “trial” (Supplementary file 1). To find relevant abstracts, we manually searched conference sessions on linkage to care and retention in care at AIDS and IAS conferences from 2008 to 2015 and CROI 2014 and 2015 (CROI abstracts from earlier years are not available). To identify sources missed by these methods, we searched reference lists of review articles identified through electronic database searches. PubMed was also searched to determine if conference abstracts have been published as full articles.

We then screened the articles that met the criteria for inclusion in the larger review for results pertaining to ART initiation, sub-Saharan Africa and adults. Finally, we updated the search for publications between 1 March 2015 and 25 May 2016 using a targeted search strategy focusing on initiation, using the search syntax [(HIV OR “antiretroviral therapy”) AND (initiation) AND (efficacy OR evaluation OR intervention OR trial) AND (Africa)] in PubMed and manually searched abstracts presented at IAS 2015 and CROI 2016.

MPF conducted the primary search and SR conducted the targeted search. After excluding those whose titles were not relevant, abstracts were read to determine eligibility. Full-text articles were reviewed by both authors to confirm eligibility. Uncertainties were resolved through consensus of both authors. We did not contact the authors of studies for primary data.

### Analysis

After extracting a standard set of indicators from each article, we first described the interventions included as to country, population, intervention, dates and outcomes. We then grouped the interventions by major approach into seven categories: counselling and support, HIV/TB integration, interventions within provider-initiated HIV testing, home-based HIV self-testing, use of a rapid/point-of-care (POC) CD4 count, improved clinic operations and implementing a package of patient-centred services. Where an intervention could arguably be assigned to more than one category, we chose the one that captured the aspect of the intervention most emphasized by the authors. Although the categories represent roughly similar approaches, interventions within categories and the methods used to evaluate them were heterogeneous, and category results should be interpreted with caution. By category, we estimated the measure of effect for each study with corresponding 95% confidence intervals (CIs) as reported, or when not reported, as calculated from the data.

We assessed the quality of the body of studies in each category of interventions using the GRADE methodology [7]. We noted that because many of the studies reviewed were observational in nature, few were expected to be considered high quality using the GRADE methodology. We then conducted a random-effects meta-analysis for each category to estimate a summary relative risk (RR) and 95% CI for each category of interventions. For each, we present the results, relative weights and the corresponding I^2^ values.

## Results

**Figure 1:**
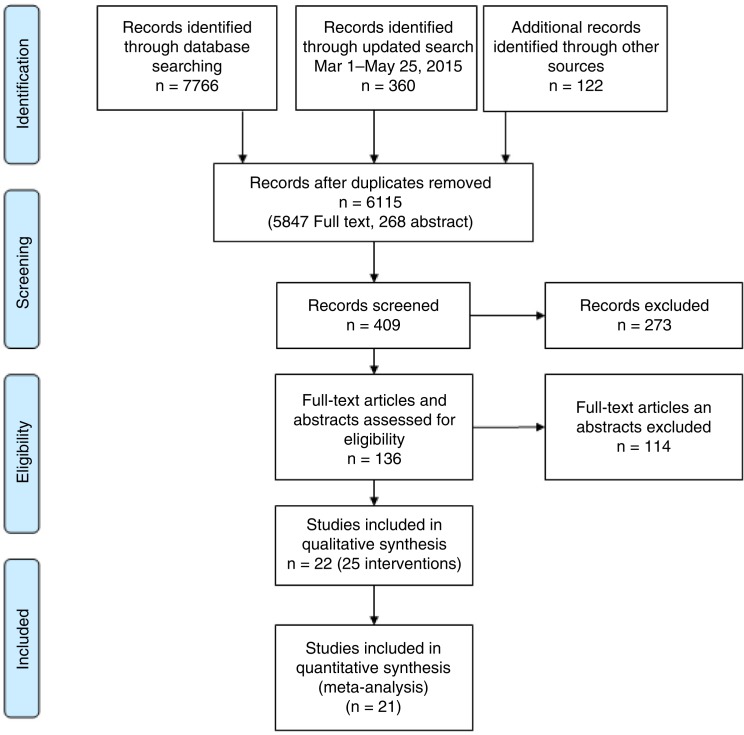
Flow chart of included and excluded studies (as per PRISMA).

Our primary search, illustrated in Figure 1, identified a total of 8248 full-text articles and abstracts. After an initial screen of the titles and abstracts, 409 citations met our initial screening criteria. Upon further review, 136 were deemed relevant for full-text review. Of these, 22 met all the inclusion criteria and were included in the final review.

The 22 included studies, which evaluated 25 interventions, are described in Table 1. They included data on 45,393 individual patients. Nine countries were represented, all in eastern or southern Africa. Three studies enrolled both adult and paediatric patients as defined by the studies, while the rest enrolled only adults. All of the studies were published in 2010 or later, with a large proportion (55%, 12/22) published in 2014 or later, signalling a recent rise in attention to this issue. About 55% (12/22) of the studies included were observational in nature with either pre-post (36%, 8/22) or parallel (18%, 4/22) designs. The remaining 45% (10/22) were randomized trials, primarily individually randomized trials (32%, 7/22).

**Table 1:** Characteristics of studies included in the review

Publication			Location		Study				
		
Study ID	Type	Year	Country	Sites	Design	Population	Data collection	Starting point	Ending point
**Counselling/support**									
Barnabas 1 [8]	Article	2016	South Africa and Uganda	KwaZulu-Natal and Sheema Districts	Individually randomized trial	HIV-positive patients at home-based testing	June 2013 to Feb 2015	HIV testing	ART initiation
Barnabas 2 [8]	Article	2016	South Africa and Uganda	KwaZulu-Natal and Sheema Districts	Individually randomized trial	HIV-positive patients at home-based testing	June 2013 to Feb 2015	HIV testing	ART initiation
Bassett [9]	Abstract	2015	South Africa	Two hospital outpatient departments and two primary health clinics	Individually randomized trial	Adults newly testing HIV positive and ART eligible		HIV testing	On ART three months
Chang [10]	Article	2015	Uganda	Rakai District	Individually randomized trial	Adults HIV positive not on ART	June 2011 to July 2013	HIV testing	ART initiation
Faal 1 [11]	Article	2011	South Africa	One urban primary healthcare clinic (Esselen clinic) in the inner city of Johannesburg	Individually randomized trial	Adults newly testing HIV positive and ART eligible	Aug to Dec 2009	HIV testing	ART initiation
**HIV/TB integration**									
Louwagie [12]	Article	2012	South Africa	46 TB treatment points in Tshwane, South Africa	Cohort study	Adults HIV positive with TB who were ART eligible	Oct 2008 to Mar 2009 (enrolment)	HIV testing	ART initiation
Hermans [13]	Article	2012	Uganda	The Infectious Diseases Institute at Makerere, University College of Health Sciences in Kampala, Uganda	Pre/post cohort study	Adults HIV positive with TB	2007 and 2009	TB treatment initiation	ART initiation
Van Rie [14]	Article	2014	Democratic Republic of Congo (DRC)	5 clinics in Kinshasa, DRC	Pre/post cohort study	Adults HIV positive with TB	Jan 2006 to Nov 2009	HIV testing	ART initiation
**Provider-initiated counselling and testing**									
Clouse [15]	Article	2014	South Africa	Witkoppen Health and Wellness Centre	Pre/post cohort study	Adults newly testing HIV positive and ART eligible	Jan 2010 to July 2012 (enrolment)	HIV testing	ART initiation
Topp [16]	Article	2012	Zambia	Seven urban-integrated primary care clinics	Cohort study	Adults and children newly testing HIV positive and ART eligible	July 2008 to June 2011	HIV testing	ART initiation
**Interventions within home-based HIV testing**									
Desai [17]	Abstract	2015	Kenya	2 rural districts of Western Kenya	Cluster-randomized trial	Adults newly testing HIV positive	July 2013 to Feb 2014 (enrolment)	HIV testing	ART initiation
MacPherson [18]	Article	2014	Malawi	Multiple sites in Blantyre, Malawi	Cluster-randomized trial	Adults (all) in the study clusters	Jan 30 to Nov 5, 2012	HIV testing	ART initiation
**Rapid/point-of-care CD4 count technology**									
Barnabas 3 [8]	Article	2016	South Africa and Uganda	KwaZulu-Natal and Sheema Districts	Individually randomized trial	HIV-positive patients at home-based testing	June 2013 to Feb 2015	HIV testing	ART initiation
Faal 2 [11]	Article	2011	South Africa	One urban primary health care clinic (Esselen clinic) in the inner city of Johannesburg	Individually randomized trial	Adults newly testing HIV positive and ART eligible	Aug to Dec 2009	HIV testing	ART initiation
Jani [19]	Article	2011	Mozambique	Four public primary health clinics in the Maputo and Sofala provinces	Cohort study	Enrolled adults and children getting a blood draw for CD4 staging	2009	CD4 staging completion	ART initiation
Nicholas [20]	Abstract	2015	Malawi	Rural decentralized health centres in Chiradzulu District, Malawi	Cohort study	Adults and children	July 2013 to Oct 2014	CD4 blood draw	ART initiation
Larson [21]	Article	2013	South Africa	Themba Lethu Clinic, Johannesburg	Pre/post cohort study	Adults newly testing HIV positive	Jan 2008 to July 2010	HIV testing	ART initiation
Matambo [22]	Abstract	2012	South Africa	Musina Sub-District	Pre/post cohort study	Adults newly testing HIV positive	July 2009 to Dec 2011	HIV testing	ART initiation
Moyo [23]	Abstract	2015	Botswana	Six rural clinics in Tutume	Pre/post cohort study		Jan 2013 to Feb 2014		ART initiation
**Improved clinic operations**									
Fairall [24]	Article	2012	South Africa	31 primary care clinics in the Free State Province	Cluster-randomized trial	Adults HIV positive not on ART but eligible or approaching eligibility	Jan 28, 2008 to June 30, 2010	CD4 staging completion	ART initiation
Pfeiffer [25]	Article	2010	Mozambique	12 clinics in Sofala and Manica Provinces	Pre/post cohort study	Adults eligible for ART	2004 to 2007	ART eligibility	ART initiation
Rosen [26]	Article	2016	South Africa	Two public sector outpatient clinics in Johannesburg	Individually randomized trial	Adults newly testing HIV positive	Apr 2013 to Aug 2014 (enrolment)	HIV testing	ART initiation
**Package of patient services**									
Burtle [27]	Article	2012	Swaziland	Good Shepherd Hospital, the district referral hospital for the Lubombo region	Pre/post cohort study	Adults eligible for ART	Feb 2009 to Feb 2010 (enrolment)	ART eligibility	ART initiation
Siedner [28]	Article	2015	Uganda	Mbarara, Uganda	Pre/post cohort study	Adults HIV positive	Jan 2012 to Nov 2013	CD4 blood draw	ART initiation
Wanyenze [29]	Article	2013	Uganda	Mulago Hospital, Uganda	Individually randomized trial	Adults newly testing HIV positive and ART eligible	May 2008 to June 2011 (enrolment)	ART eligibility	ART initiation

ART initiation indicates that at least the first dose of ARV medications has been prescribed or dispensed.

**Figure 2:**
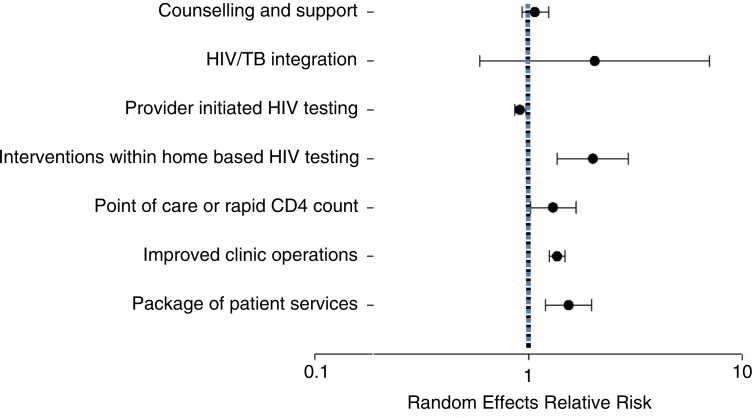
Summary relative risks from a random effects meta-analysis of data from each category of intervention.

The interventions evaluated in each study, the outcomes assessed and the results as grouped by the authors are presented in Table 2. Table 3 presents our assessment of the quality of evidence and meta-analysis results by category of intervention. Below, we summarize results for each category of interventions and the evidence for each as shown in Tables 1–3. Effects of all the interventions are synthesized using a random effect meta-analysis in Figure 2. Forest plots for each set of interventions with corresponding weights and I^2^ values are given in Supplementary file 2.

**Table 2:** Reported results of included studies

Study ID	Intervention	Comparison	Outcome	Timing of outcome	*N* intervention (control)	Risk/rate intervention (control)	Effect size	95% confidence interval	*p*	Interpretation
Counselling and support										
Barnabas 1	Clinic visit facilitation	Standard of care referral	ART initiation		431 (423)	0.37 (0.34)	RR 1.11	0.92–1.34	0.26	Clinic visit facilitation was not associated with any difference in ART initiation
Barnabas 2	Lay counsellor home follow-up	Standard of care referral	ART initiation		449 (423)	0.41 (0.34)	RR 1.23	1.02–1.47	0.028	Lay counsellor follow-up was associated with an increase in ART initiation
Bassett	Patient navigators using a strengths-based case-management approach and scheduled phone calls and text messages over four months	Standard of care	On ART for those ART eligible	Three months on ART	618 (528)	0.34 (0.37)	RR 0.92^a^	0.79–1.07^a^	0.6	This approach to patient navigation was not associated with an increase in linkage to care
Chang	Peer supporters with monthly visits to provide support and counselling	Standard of care	Currently on ART	One year	216 (215)	0.32 (0.30)	RR 1.09	0.81–1.45		This approach to peer support was not associated with an increase in treatment initiation
Faal 1	Immediate receipt of CD4 count results (FACSCount)	Standard collection of CD4 result only	ART initiation	One month	35 (36)	0.37 (0.25)	RR 1.49^a^	0.37–3.03^a^		Leaflets were not associated with a significant increase in ART initiation among those ART eligible.
**HIV/TB integration**										
Hermans	Integrated TB/HIV care and treatment	Standard of care	ART initiation		243 (228)	0.57 (0.66)	RR 0.86	0.75–1.0^a^	0.034	ART and TB treatment integration did not lead to an increase in ART initiation
Louwagie	ART and TB care at same site (“semi-integrated”)	Geographically separately rendered HIV and TB care	ART initiation		105 (233)	0.71 (0.45)	sHR 2.49	1.06–5.88		ART and TB treatment under one roof was associated with an increase in ART initiation for HIV-positive TB patients
Van Rie	Integrated TB/HIV care and treatment	Standard of care referral to centralized ART facility after diagnosis	ART initiation		513 (373)	0.69 (0.17)	RR 4.06^a^	3.21–5.13^a^		Integrated services was associated with an increase in ART initiation
**Provider-initiated HIV testing**										
Clouse	Systematic opt-out HCT for all adult clients	Targeted PICT and voluntary counselling and testing	ART initiation	Twelve months after diagnosis	717 (744)	0.64 (0.59)	RR 1.08^a^	1.00–1.18^a^	0.05	Systematic opt-out HCT was associated with a small increase in ART initiation among those ART eligible
Topp	Provider-initiated testing and counselling for adults and children	Voluntary counselling and testing	ART initiation		1655 (6520)	0.72 (0.69)	aOR 0.9	0.82–0.97	0.01	Integrated care was associated with a small decrease in the odds of being initiated on ART if eligible
**Interventions combined with home-based HIV testing**										
Desai	POC CD4 count at home-based HIV testing with referral	Standard of care home-based HIV testing and referral	ART initiation		371 (321)	0.17 (0.10)	RR 1.65^a^	1.11–2.54^a^	0.01	POC CD4 during home-based HCT was associated with an increase in ART initiation
MacPherson	HIV self-testing followed by optional home initiation of HIV care	HIV self-testing accompanied by facility-based HIV care	ART initiation	Six months	8194 (8466)	0.022 (0.007)	aRR 2.44	1.61–3.68	<0.001	HIV self-testing followed by optional home initiation was associated with a significant increase in ART initiation over six months among all testers
**Rapid/Point-of-care CD4 count**										
Barnabas 3	POC CD4 count (Pima)	Standard of care referral	ART initiation		627 (676)	0.39 (0.36)	RR 1.08	0.94–1.26	0.28	POC CD4 count staging was not associated with a significant increase in ART initiation
Faal 2	Same day CD4 count results (FACSCount)	Standard collection of CD4 result only	ART initiation	One month	43 (36)	0.65 (0.25)	RR 2.1	1.39–3.17		Same day receipt of CD4 counts was associated with a significant increase in ART initiation among those ART eligible.
Jani	POC CD4 count (Pima)	Standard of care lab referral of blood for CD4 staging	ART initiation		437 (492)	0.65 (0.61)	OR 1.07^a^	0.87–1.30^a^		POC CD4 count staging was not associated with a significant increase in ART initiation among those eligible
Larson	Same day CD4 count results (FACSCount)	Standard of care	ART initiation	≤16 weeks	273 (223)	0.49 (0.46)	aRR 1.2	0.99–1.46	0.06	Rapid POC CD4 results were associated with a small non-significant increase in ART initiation among eligible
Matambo	Integrated mobile HIV/TB primary health care with POC CD4 testing (Pima)	Standard of care	ART initiation		226 (380)	0.83 (0.51)	RR 1.63^a^	1.45–1.83^a^	<0.0001	Integrated services was associated with an increase in linkage to care
Moyo	Point-of-care CD4 count (Pima)	Standard of care	ART initiation				RR 1.33		0.01	POC led to an increase in ART initiation
Nicholas	Point-of-care CD4 count (Pima)	Standard of care	ART initiation	Any time	253 (259)		RR 0.96	0.91–1.01		POC led to no overall increase in ART initiation among those eligible
**Improved clinic operations**										
Fairall	Prescribing nurses given educational outreach training sessions about ART prescribing and task shifting to nurses	Standard of care	ART initiation	At least 12 months	5390 (3862)	0.69 (0.63)	RR 1.24^b^	0.88–1.73	0.218	Training and task shifting to nurses was associated with a small non-significant increase in ART initiation
Pfeiffer	HIV service integration including co-location of services; training personnel to provide multiple services; training to link separate services; strengthening linkages between facility levels; and harmonization of data collection	Standard of care	ART initiation	≤90 days of eligibility			RR 1.58	1.17–2.14		HIV service integration was associated with an increase in ART initiation
Rosen	Immediate (rapid) ART initiation including POC technology and service delivery acceleration	Standard of care	ART initiation	≤90 days after testing HIV positive and ART eligible	187 (190)	0.97 (0.72)	RR 1.36	1.25–1.49		Immediate ART initiation was associated with an increase in uptake of ART within 90 days
**Package of patient services**										
Burtle	Introduction of pre-ART interventions, including task shifting, counselling, clinical staging, timely ART initiation, social and psychological support	Standard of care	ART initiation		419 (68)	0.81 (0.53)	RR 1.53^a^	1.22–1.92^a^		The intervention was associated with a 50% increase in ART initiation among those ART eligible
Siedner	SMS notifying patients of CD4 results; if early return to clinic required, one of three messages and transport reimbursement	Standard of care	ART initiation		110 (26)	0.96 (0.81)	aHR 2.26	1.38–3.73	0.001	SMS notification was associated with a significant increase in ART initiation
Wanyenze	Enhanced linkage with case-management referral (counselling, assisted disclosure of HIV status, staff introduction and scheduling, reminder via telephone or home visit one week before the scheduled appointment) and tracing of lost patients	Standard linkage to care (explanation of services, hours, and locations of the clinics nearby)	ART initiation among those eligible	One year	202 (183)	0.78 (0.71)	aHR 1.29^c^	1.03–1.67^c^	0.03	Enhanced linkage was associated with a significant increase in ART initiation among those eligible

RR, relative risk; aRR, adjusted relative risk; aIRR, adjusted incidence rate ratio; OR, odds ratio; aOR, adjusted odds ratio; aHR, adjusted hazard ratio; PR, prevalence ratio.

a Relative risk and 95% CI not reported but approximated from the data.

b Adjusted for clustering.

c Presenting the invers of the results (i.e. 1/(results presented)) as the comparison provided was the effect of standard of care vs. intervention.

**Table 3:** GRADE quality assessment and random effects meta-analysis of categories of interventions to improve ART initiation

	Risk of:							
					
# (type) studies	Bias	Inconsistency	Indirectness	Imprecision	*N* intervention (control)	Risk intervention (control)	Random effects meta-analysis relative risk (95% CI)	Quality
**Counselling and support**								
5 (5 iRCT)^a^	Not serious	Not serious	Not serious	Not serious	1749 (1202)	0.34 (0.32)	1.08 (0.94–1.26)	Moderate^b^
**HIV/TB integration**								
3 (2 pre/post, 1 cohort)	Serious	Not serious	Serious	Not serious	846 (849)	0.66 (0.39)	2.05 (0.59–7.09)	Very low^c^
**Provider-initiated HIV testing**								
2 (1 cohort, 1 pre/post)	Serious	Serious	Serious	Not serious	2399 (7237)	0.68 (0.69)	0.91 (0.86–0.97)	Very low^c^
**Interventions combined with home-based HIV testing**								
2 (2 cRCT)	Not serious	Not serious	Not serious	Serious	8565 (8787)	0.03 (0.01)	2.00 (1.36–2.92)	Low^c^
**Rapid/point-of-care CD4 count technology**								
7 (3 pre/post, 2 cohort, 2 iRCT)^d^	Serious	Serious	Serious	Not serious	1524 (1598)	0.50 (0.42)	1.26 (1.02–1.55)	Low
**Improved clinic operations**								
3 (1 iRCT, 1 cRCT, 1 pre/post)	Serious	Not serious	Not serious	Not serious	5577 (4052)	0.70 (0.63)	1.36 (1.25–1.48)	Low^b^
**Package of patient services**								
3 (1 iRCT, 2 pre/post)	Serious	Not serious	Serious	Not serious	731 (277)	0.80 (0.63)	1.54 (1.20–1.97)	Low^b^

cRCT, cluster-randomized trial; iRCT, individually randomized trial.

a Four interventions from three studies. As the same control was used for comparison to both interventions in Barnabas 2015, we did not double count the control group in the total control subjects.

b Graded down one level as few studies.

c Graded down two levels as few studies and risk of bias.

d One study (Moyo) not included in meta-analysis as no Ns provided and no variance provided.

### Counselling and support interventions

We identified five counselling and support interventions evaluated in four studies, all conducted in South Africa and/or Uganda. In total, the studies included 2951 individuals. All were individually randomized trials. The interventions were lay counsellor home visits after a home-based CD4 count [8]; lay counsellor clinic visit facilitation [8]; home visits, calls and text messages by patient navigators [9]; home visits by peer supporters [10]; and provision of an informational brochure to patients explaining how to obtain further care [11]. Rates of ART initiation among control-arm patients eligible for treatment were low, at only 32% when pooled across the four studies. Only one of the four interventions, lay counsellor home visits, had a significant positive effect with a risk difference of 7% (RR [95% CI] 1.23 [1.03–1.46]) [8]. Our meta-analysis estimated that the counselling and support interventions included in the review had little to no impact on ART initiation (RR 1.08; 95% CI: 0.94–1.26). Because all four studies were randomized trials, this was the intervention category with the overall best quality and was graded as moderate quality, as shown in Table 3.

### HIV/TB integration

We found three studies that reported on interventions to integrate HIV and TB services. One was a cohort study in South Africa that examined co-locating HIV and TB services (referred to as “semi-integrated”) [12], while two were pre–post studies of fully integrated HIV and TB services, one in Uganda [13] and one in the Democratic Republic of Congo [14]. In total, the three studies included 1695 subjects. Two of the three studies showed a large benefit. When pooled across the three studies, rates of ART initiation were moderate in the control arm (39%). When combined in a meta-analysis, HIV/TB service integration was associated with twofold increase in ART initiation compared to non-integrated care (RR: 2.05; 95% CI: 0.59–7.09) but with very poor precision. With only three studies, all of which were observational, the overall quality of evidence was very low.

### Provider-initiated HIV testing

Two studies reported the impact of Provider-Initiated HIV Counseling and Testing (PITC) on ART initiation, one in South Africa [15] and one in Zambia [16]. One was a pre–post study and one was a cohort study. They included a total of 9636 subjects. One of the interventions showed a very small increase and the other a decrease in ART initiation associated with PITC. Overall ART initiation in the control group was 69%. When the data were combined through meta-analysis, PITC was the only category of interventions that was associated with reduced ART initiation (RR: 0.91; 95% CI: 0.86–0.97). This should be interpreted with caution as the absolute reduction in ART initiation was only 1%. In addition, it is important to note here that patients in PITC and those identified through Voluntary Counseling and Testing (VCT) are likely different with respect to their disease stage, making it difficult to draw strong conclusions. As there were only two studies and all were observational, the overall quality of the evidence was very low.

### Interventions combined with home-based HIV testing

Two cluster-randomized trials examined the effect on ART initiation of interventions combined with home-based HIV testing, one in Kenya and one in Malawi. The study in Kenya compared home-based testing with POC CD4 counts to home-based testing with standard referral [17]. The study in Malawi compared home-based testing with optional home ART initiation to home-based testing with facility-based care [18]. Both showed a benefit in terms of ART initiation. In our meta-analysis, home-based testing was associated with an increase in ART initiation (RR: 2.00; 95% CI: 1.36–2.92). The studies included a total of 17,352 subjects, but it is important to note that the denominator in the Malawi study [18] included all persons tested, not just those testing HIV positive or eligible for ART, making overall rates of ART initiation appear very low. This has a strong influence on the overall results, as the pooled rate of ART initiation in the control group was just 0.7%, leading the meta-analysis relative estimate of a 100% increase in ART initiation to translate into only a 1.7% absolute increase. While both of the studies included were randomized controlled trial (RCTs), as there were only two studies, the overall quality of the evidence was graded as low.

### Rapid POC CD4 count technology

Five observational studies and two randomized trials evaluated the effect of rapid/POC CD4 count technology on ART initiation in South Africa (4), Botswana, Malawi, Mozambique and Uganda. Five tested the effect of POC testing using Alere Pima machines [8,19,20,22,29] and two used same day BD FACS Count results [11,21]. One study did not report the sample size or a CI for its reported RR so could not be included in the meta-analysis [22]. The remaining six studies enrolled 3122 subjects. The pooled rate of ART initiation in the control group was 42%. Three of the interventions showed a benefit, while three showed little or no effect. In our meta-analysis, rapid/POC CD4 count technology was associated with an increase in ART initiation (RR: 1.26; 95% CI: 1.02–1.55). While this category had the largest number of studies, all but two were observational, and the overall quality of the evidence was thus considered low.

### Improved clinic operations

Two studies conducted in South Africa [23,24] and one in Mozambique [25] evaluated multi-faceted changes to clinic operations. The interventions, which were very diverse, are described in detail in Tables 1 and 2. Each included two or more of a range of activities: enhanced counselling and support, task shifting, provider training, POC technology, HIV service integration, improved clinic management and others. Two were RCTs (one individually randomized, one cluster randomized) and one was an observational study. One study did not report the sample size [25], but for the two remaining, the total number of subjects was 9629. The pooled rate of ART initiation in the control group was 63%. All three of the interventions increased ART initiation (though one had a very wide CI) [23]. In our meta-analysis, improved clinic operations showed a benefit in terms of increased ART initiation (RR: 1.36; 95% CI: 1.25–1.48). This result should be interpreted with caution, however, given the heterogeneity of the interventions. The specific mix of activities included in each intervention may determine its effect, such that different combinations would produce different results from those reported here. Overall, the quality of the evidence was low, as there were only three studies and one was a pre–post design.

### Package of patient services

We identified three studies that explored the impact of a package of patient-directed services. As with the previous category, each package included two or more services, described in detail in Tables 1 and 2. One was a pre–post study in Swaziland [26] that tested the effect of a package of pre-ART services; the second, a pre–post study in Uganda that tested the effect of SMS notification of CD4 results combined with transport reimbursement [27]; and the third, a randomized trial in Uganda [28] that tested a package of enhanced linkage with case-management referral. All three showed a benefit in terms of treatment initiation. The rate of ART initiation in the control group was 63%. In our meta-analysis, the interventions were associated with an increase in initiation (RR: 1.54; 95% CI: 1.20–1.97). As there were only three studies and two were observational, the evidence was considered low quality.

## Discussion

Over the decade of large-scale public sector access to HIV treatment in sub-Saharan Africa, numerous reviews have documented losses from the HIV care and treatment cascade [30], documenting particularly high attrition between HIV testing and ART initiation [2]. In the light of the World Health Organization's recent recommendation that treatment be offered to all people living with HIV at any CD4 count [31], the steps needed for patients to access care will change dramatically, effectively eliminating the interval of “pre-ART care” during which ineligible patients were monitored for disease progression. In the new cascade of care that the WHO recommendations suggest, the most likely points at which patients who test positive for HIV will become lost to care are between linkages from an HIV testing site to an HIV treatment site and between an initial visit to an HIV treatment site and ART initiation. To inform this new paradigm, we systematically reviewed the literature on interventions aimed at pre-ART care but which specifically focused on, or presented data on, changes in the rate or timing of ART initiation, the outcome most relevant to this new, simplified cascade of care. We focused on sub-Saharan Africa as most of the studies we identified overall were from this region, making it difficult to generalize to other areas.

Because interventions to improve pre-ART care outcomes are diverse and heterogeneous, we grouped the interventions into categories representing similar approaches to improving care. In interpreting the results presented here, we reiterate that both the interventions included in each category and the methods used to evaluate them differed widely, such that any conclusions drawn should be treated with caution.

While the overall body of evidence was mixed, we found several approaches that were promising in terms of ART initiation. Integrating HIV and TB services, whether through simply co-locating the services, or fully integrating them, was associated with a roughly twofold increase in rates of ART initiation among individuals with active TB and living with HIV. This finding expands upon the conclusions of a previous 2011 review [32], which found benefits from co-locating services for adherence and retention on ART but provided little evidence on whether co-location improved ART uptake. As our results are based on only three studies and low-quality evidence, and one of the three studies did not find a benefit for ART initiation, more research on TB/HIV integration interventions is needed before strong conclusions can be drawn.

Another area that showed promise was the use of rapid and/or POC CD4 count technology. Use of machines such as the Alere Pima for rapid CD4 results increases the proportion of patients who learn that they are eligible for treatment and reduces the number of visits required to initiate treatment [33]. As the most recent WHO recommendation to initiate ART regardless of CD4 count is adopted into national policies, the CD4 count will lose its primary role in establishing treatment eligibility. Nonetheless, CD4 counts may be retained as a valuable clinical component of the initiation algorithm in many countries for years to come, in identification of the sickest. In the studies reviewed here, use of rapid and/or POC CD4 count technology was associated with about a 25% increase in ART initiation (random effects RR 1.26; 95% CI: 1.02–1.55) compared to standard of care referral for CD4 testing. Offering a POC CD4 count has previously been shown to be effective at increasing the proportion of patients who receive their CD4 test results [33–35]. Our findings with regard to ART initiation are in the same direction as, but smaller than, those of a previous meta-analysis on POC CD4 testing, which reported a RR of 1.8 (95% CI: 1.1–2.9) [34].

Finally, multi-faceted interventions that improved clinic operations or offered a package of patient services also showed promise and perhaps have the most relevance to future treatment guidelines. Such approaches target more than one step in the cascade, strengthening both linkage to care after HIV testing and treatment initiation after linkage. In these two categories, we found only five studies in total, but all reported a benefit, with a combined risk ratio of 1.36 (95% CI: 1.25–1.48) for improved clinic operations and 1.54 (95% CI: 1.20–2.00) for improvements in the package of patient services. Although these results agree with data from other parts of the world and in other patient populations, the approaches remain diverse and the quality of the evidence is low. More high-quality studies will be needed before we draw strong conclusions and discern which specific components of the interventions might be most important for achieving results.

Other interventions, including peer and lay counsellor support and provider-initiated HIV testing, showed little impact on ART initiation. This is particularly disappointing for peer and lay counsellor support, which had previously been found to be effective at increasing linkage to care [33] but here had no benefit for ART initiation. PITC, moreover, appears to be associated with a slight reduction in ART initiation, though in the light of the low quality of evidence, at best we can say there appears to be no benefit. PITC may be identifying patients who do not wish to volunteer for care and thus increase the denominator (patients who could start ART) without changing the numerator (patients who do start ART).

In contrast to the PITC results, the studies reviewed suggest that interventions within a platform of home-based HIV testing have promise for increasing ART uptake. This category does not capture the effects of home-based testing itself but rather the effects of interventions designed to improve linkage or ART initiation after home-based testing. The two interventions included in the review were use of POC CD4 counts to determine treatment eligibility and optional home ART initiation; both were effective but further evidence will be needed for each one before general conclusions about home-based services can be drawn.

Beyond the small number of studies that estimated the effect of interventions in increasing ART uptake, the overall quality of the studies was poor. To some extent, the apparent low quality of the literature reviewed here stems from the fact that interventions to improve pre-ART care and increase uptake of ART are largely structural or behavioural. Unlike for drug trials, results depend heavily on the details of how the intervention was designed, to whom it was delivered (in terms of population age, gender, socioeconomic status), where it was delivered (community, facility level, etc.), which outcomes were assessed and to what they were compared (standard of care, another intervention, etc.). In this review, we found very few reports of studies that evaluated the effect of the same intervention on the same outcome or population. For example, a POC CD4 count using the same technology may have sharply different results in urban and rural settings or when used at community-based or facility-based HIV testing sites. For this reason, trying to generalize from just a few studies – even those of moderate or high quality – is potentially misleading. For every intervention considered, context – location, population, outcome and so on – is an essential component of understanding effectiveness.

In addition to the need for more, and more rigorous, evaluations of interventions, a consideration that was omitted from nearly all the studies reviewed here is retention of patients on ART in the immediate aftermath of initiation. For most researchers examining the pre-ART care period, ART initiation has been an endpoint with no follow-up to investigate whether the mode or timing of initiation is associated with outcomes once treatment has been started. As the global paradigm for “pre-ART care” evolves, and more effective ways to move patients from HIV testing to ART initiation are sought and implemented, studies that assess not just uptake of ART, but uptake with early retention on ART, would strengthen the evidence base.

In conclusion, in a systematic review of the literature from 2008 to mid-2016 reporting on interventions to increase rates of ART initiation in sub-Saharan Africa, we found only 22 studies. They were diverse in nature, ranging from counselling or technology interventions focused on one step in the pre-ART cascade to multi-faceted rearrangements of how care is provided. Some promising approaches were identified and merit further research on their effect and cost-effectiveness in a range of settings and populations. For all the approaches identified, however, the number of studies was small and quality mixed. In view of the new global recommendation of starting all HIV-positive individuals on ART, rather than attempting to retain those with high CD4 counts in pre-ART care, researchers must invest far more effort in identifying and evaluating how best to offer treatment initiation in a manner that is both efficient for service providers and effective for patients, without jeopardizing treatment outcomes.

## Supplementary Material

Interventions to improve the rate or timing of initiation of antiretroviral therapy for HIV in sub-Saharan Africa: meta-analyses of effectiveness
