# Supplementary material for: Interventions to improve the rate or timing of initiation of antiretroviral therapy for HIV in sub-Saharan Africa: meta-analyses of effectiveness
Source: J Int AIDS Soc. 2016 Aug 8;19(1):20888. doi: 10.7448/IAS.19.1.20888 (PMC4978859; doi:10.7448/IAS.19.1.20888)

Additional file 1. Search Strategy for Each Bibliographic Database

Database	Search
PubMed	((linkage OR pre-ART OR initiation OR retention OR attrition OR "loss to follow up") AND ((HIV OR "antiretroviral therapy")) AND ((efficacy OR evaluation OR intervention OR trial))) AND (("2008/01/01"[PDat] : "2016/12/31"[PDat]) AND Humans[Mesh] AND English[lang])
ISI Web of Knowledge	(TS=((HIV OR "antiretroviral therapy")) AND TS=((linkage OR pre-ART OR initiation OR retention OR attrition OR "loss to follow up")) AND TS=((efficacy OR evaluation OR intervention OR trial))) *AND* LANGUAGE: (English) *AND* DOCUMENT TYPES: (Article OR Proceedings Paper) Indexes=SCI-EXPANDED, SSCI, A&HCI, CPCI-S, CPCI-SSH Timespan=2008-2015
EMBASE	'hiv'/exp or hiv or 'antiretroviral therapy' and (linkage or 'pre art' or initiation or retention or attrition or 'loss to follow up') and (efficacy or 'evaluation'/exp or evaluation or intervention or trial) and [english]/lim and [abstracts]/lim and [2008-2015]/py and [embase]/lim and [humans]/lim
LILACS Portal	(HIV OR antiretroviral) AND (linkage OR pre-ART OR initiation OR retention OR attrition OR adherence OR compliance) AND (efficacy OR evaluation OR intervention OR trial) AND (db:("LILACS"))
Global Index Medicus	(HIV) AND (linkage OR retention)
African Index Medicus	(HIV) AND (linkage OR retention)

Additional file 2. Forest plots of each intervention, relative weights and corresponding I^2 values

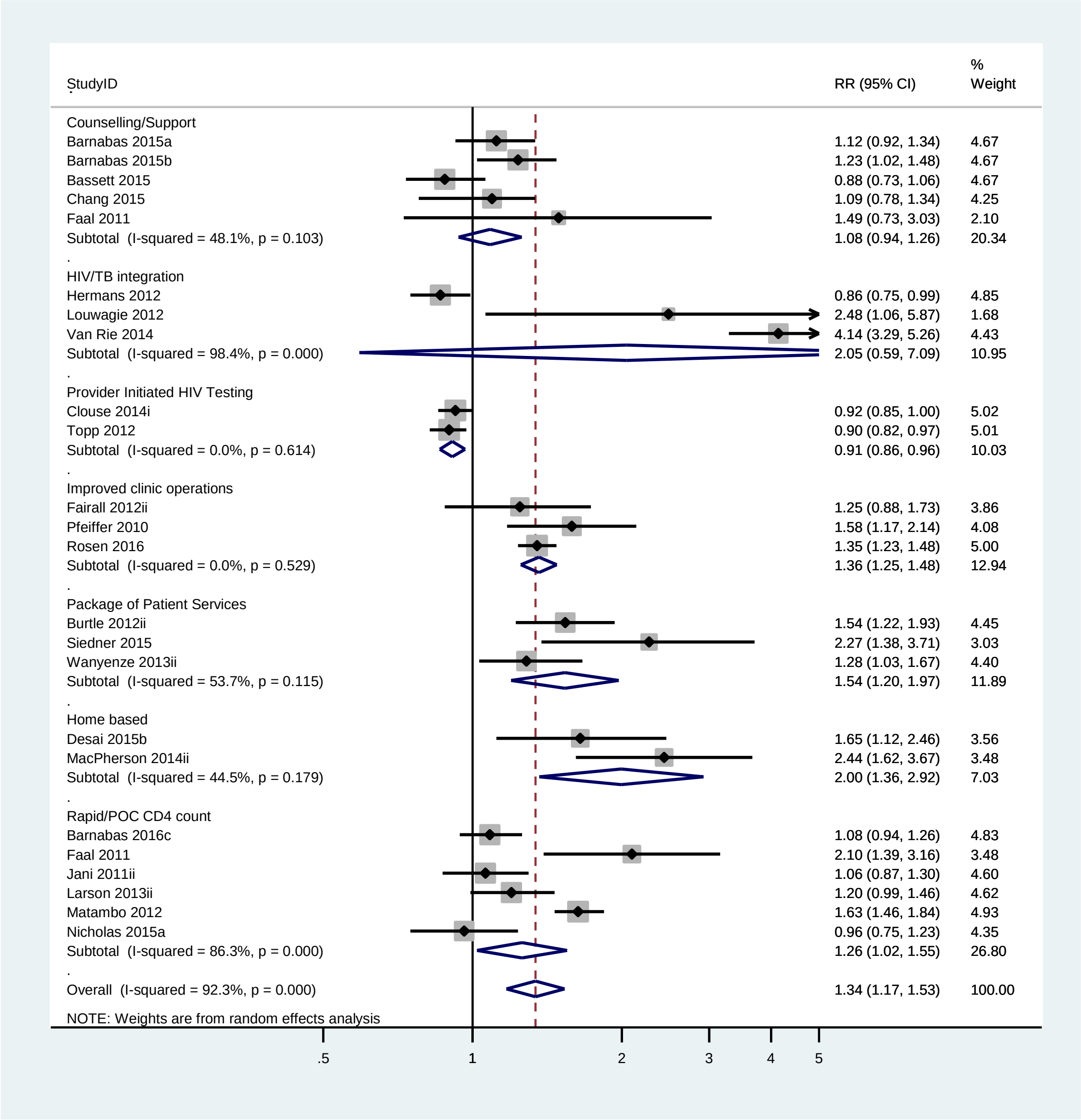

Supplement: Interventions to improve the rate or timing of initiation of antiretroviral therapy for HIV in sub-Saharan Africa: meta-analyses of effectiveness [file JIAS-19-20888-s001.pdf]
